# Efficacy of artesunate–amodiaquine in the treatment of falciparum uncomplicated malaria in Madagascar

**DOI:** 10.1186/s12936-018-2440-0

**Published:** 2018-08-06

**Authors:** Oméga Raobela, Valérie Andriantsoanirina, David Gael Rajaonera, Tovonahary Angelo Rakotomanga, Stéphane Rabearimanana, Fanomezantsoa Ralinoro, Didier Ménard, Arsène Ratsimbasoa

**Affiliations:** 1 National Malaria Control Programme of Madagascar, Androhibe, Antananarivo, Madagascar; 2 Faculty of Sciences, University of Antananarivo, Antananarivo, Madagascar; 3 Département Parasites et Insectes Vecteurs, Institut Pasteur Paris, Paris, France; 4 Faculty of Medicine, University of Antananarivo, Antananarivo, Madagascar

**Keywords:** Malaria, *Plasmodium falciparum*, Therapeutic efficacy survey, Artesunate–amodiaquine, Madagascar

## Abstract

**Background:**

Since 2006, the artemisinin-based combination therapy (ACT) are recommended to treat uncomplicated malaria including non *Plasmodium falciparum* malaria in Madagascar. Artesunate–amodiaquine (ASAQ) and artemether–lumefantrine are the first- and second-line treatment in uncomplicated falciparum malaria, respectively. No clinical drug efficacy study has been published since 2009 to assess the efficacy of these two artemisinin-based combinations in Madagascar, although the incidence of malaria cases has increased from 2010 to 2016. In this context, new data about the efficacy of the drug combinations currently used to treat malaria are needed.

**Methods:**

Therapeutic efficacy studies evaluating the efficacy of ASAQ were conducted in 2012, 2013 and 2016 among falciparum malaria-infected patients aged between 6 months and 56 years, in health centres in 6 sites representing different epidemiological patterns. The 2009 World Health Organization protocol for monitoring anti-malarial drug efficacy was followed.

**Results:**

A total of 348 enrolled patients met the inclusion criteria including 108 patients in 2012 (n = 64 for Matanga, n = 44 for Ampasipotsy), 123 patients in 2013 (n = 63 for Ankazomborona, n = 60 for Anjoma Ramartina) and 117 patients in 2016 (n = 67 for Tsaratanana, n = 50 for Antanimbary). The overall cumulative PCR-corrected day 28 cure rate was 99.70% (95% IC 98.30–99.95). No significant difference in cure rates was observed overtime: 99.02% (95% IC 94.65–99.83) in 2012; 100% (95% IC 96.8–100) in 2013 and 100% (95% IC 96.65–100) in 2016.

**Conclusion:**

The ASAQ combination remains highly effective for the treatment of uncomplicated falciparum malaria in Madagascar.

## Background

Malaria remains an important health problem in Madagascar mainly in children under 5 years of age as mentioned in the national strategy plan for malaria control in Madagascar 2013–2017 [1]. Over the past decade, the burden of malaria has fluctuated over time partly due to successes and failures of anti-malarial policy [1]. The artemisinin-based combination therapy (ACT) are recommended in Madagascar since 2006 to treat uncomplicated malaria including non-falciparum malaria. The National Malaria Control Programme (NMCP) replaced chloroquine with artesunate–amodiaquine (ASAQ) combination as first-line drug for treating uncomplicated falciparum malaria, and artemether–lumefantrine as an alternative treatment. This change was based on a study, including clinical and in vitro data that reported the complete efficacy of the ASAQ combination in Madagascar [2, 3]. An additional study performed 1 year later, which was part of a multicentre trial conducted in 2006 in 5 African sites, reported similar high levels of cure rates to ASAQ in Tsiroanomandidy [4]. Since then, no data about the efficacy of ASAQ or alternative anti-malarials in Madagascar have been reported.

The incidence of malaria cases has increased in Madagascar (9.83 cases for 1000 inhabitants in 2010 to 19.52 in 2016) (NMCP data, pers. comm.). In this context, it is of utmost importance to acquire new data from recent efficacy studies. This study present the results of studies conducted from 2012 to 2016 to assess the efficacy of the ASAQ combination for the treatment of uncomplicated malaria cases in 6 sites covering three epidemiological patterns in Madagascar.

## Methods

### Study areas

**Fig. 1:**
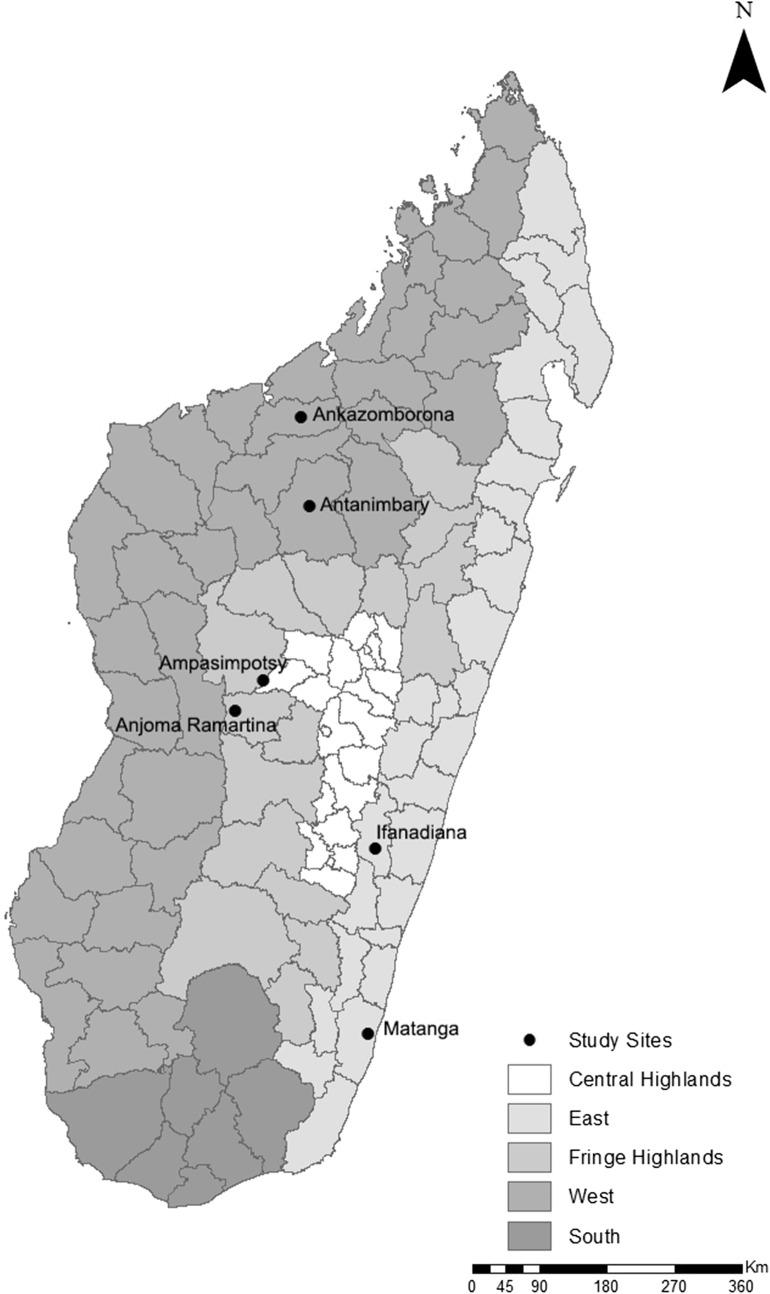
Map of Madagascar showing study sites in six districts

Clinical ASAQ efficacy studies were carried out in 6 sites belonging to 3 epidemiological strata, from May to September 2012 in Matanga (Vangaindrano) and Ampasimpotsy (Tsiroanomandidy), from March to May 2013 in Ankazomborona (Marovoay) and Anjoma Ramartina (Mandoto), and from March to June 2016 in Tsaratanana (Ifanadiana) and Antanimbary (Maevatanana) (Fig. 1).

Matanga and Tsaratanana are located in eastern Madagascar (equatorial stratum, perennial and endemic area), Antanimbary (Maevatanana) and Ankazomborona (Marovoay) in western Madagascar (tropical stratum, seasonal and endemic area) and Ampasimpotsy (Tsiroanomandidy) and Anjoma Ramartina (Mandoto) in the fringe highlands of Madagascar (fringe highlands stratum, seasonal and low endemic area).

### Patient recruitment

Clinical ASAQ efficacy studies were conducted according to 2009 World Health Organization protocol for monitoring anti-malarial drug efficacy [5]. Febrile patients or patients with fever history seeking anti-malarial treatment in health centres were screened for malaria by rapid diagnostic test (SD Bioline Ag P.f/Pan, Standard Diagnostics INC, Korea). Positive cases for *Plasmodium falciparum* were enrolled if they met inclusion criteria and gave their written informed consent. Inclusion criteria were: (i) age > 6 months; (ii) axillary temperature ≥ 37.5 °C or history of fever in the 24 h preceding consultation; (iii) *P. falciparum* mono-infection with parasitaemia between 1000 and 200,000 asexual parasites per µl; and, (iv) absence of signs of severe malaria. Children were recruited after parental consent.

Finger-prick capillary blood sample were collected to prepare thin and thick blood smears for malaria microscopy examination, haemoglobin level determination (HemoCue HB 201, HemoCueAB, Angelholm, Sweden) and dried blood spot (DBS) for molecular biology investigations.

Artesunate–amodiaquine Winthrop^®^ (Sanofi, France) was administered daily for 3 days at the dose recommended by the manufacturer on a mg per weight basis (4.5–8 kg: 25 mg/67.5 mg tablets; 9–17.9 kg: 50 mg/135 mg tablets; 18–35 kg: 100 mg/270 mg tablets; > 36 kg: 100 mg/270 mg tablets). Participants were observed by the medical team for 30 min after treatment to monitor for vomiting or other adverse events; those who vomited were administered a second dose and observed for an additional 30 min. Patients who vomited both doses were excluded from the study and referred to the hospital for parenteral treatment.

### Patient follow-up

Enrolled patients were followed daily (days 1, 2, 3) and weekly (days 7, 14, 21, and 28). At each visit, patients were clinically examined by a physician and clinical parameters were recorded on a Case Record Form (CRF). Parasite densities estimated by microscopic examination were checked at each visit. All blood films were read by 2 qualified microscopists, and by a third independent microscopist if discordance was > 20%. Dried blood spot obtained at day 0 and day failure were used for PCR genotyping. Haemoglobin concentrations were assessed on day 0 and day 28.

### DNA extraction and molecular investigations

Parasite DNA extracted from dried blood spot (DBS) with QIAamp DNA blood kit according to manufacturer’s instruction, was amplified for confirming *Plasmodium* species with slight modifications [6] and for distinguishing recrudescence and re-infection by using *msp1* and *msp2* polymorphisms [7] in case of recrudescence.

### Data analysis

Data were entered into the standard pre-programmed Excel worksheet provided by the Global Malaria Programme WHO for per-protocol analysis. Per protocol analysis was used to assess treatment outcomes at day 28 based on the WHO 2009 criteria: ACPR (adequate clinical and parasitological response), ETF (early treatment failure), LCF (late clinical failure), and LPF (late parasitological failure).

## Results

A total of 348 patients met the inclusion criteria and were enrolled for the analyses including 108 patients in 2012 (n = 64 for Matanga, n = 44 for Ampasipotsy), 123 patients in 2013 (n = 63 for Ankazomborona, n = 60 for Anjoma Ramartina) and 117 patients in 2016 (n = 67 for Tsaratanana, n = 50 for Antanimbary). Their baseline characteristics are shown in Table 1. Ages ranged from 6 months to 56 years (median 8 years).

**Table 1:** Baseline clinical and parasitological characteristics of enrolled patients in the 6 sites, Madagascar, 2012–2016, ASAQ

Epidemiological strata	Eastern Madagascar (equatorial stratum)		Western Madagascar (tropical stratum)		Fringe highlands of Madagascar (fringe highlands stratum)	
Site	Matanga	Tsaratanana	Ankazomborona	Antanimbary	Ampasipotsy	Anjoma Ramartina
District	Vangaindrano	Ifanadiana	Marovoay	Maevatanana	Tsiroanomandidy	Mandoto
Year	2012	2016	2013	2016	2012	2013
n	64	67	63	50	44	60
Age years, median (range)	2.17 (1–34)	6 (1–14)	9 (1–54)	11 (1–56)	174 (2–56)	10 (2–45)
Gender male, n (%)	35 (54,69)	24 (35,82)	29 (46,03)	28 (35,82)	27 (61,36)	30 (50)
Axillary T°, mean (SD)	38.05 (1.38)	38.42 (1.02)	37.83 (1.46)	38.38 (1.29)	37.91 (1.14)	37.96 (1.31)
Parasite density/µl	20,803.67	28,089.02	21,517.67	22,301.17	7315.71	10,704.84
Parasite density (geometric mean, range)	1005–199,800	1561–185,103	1448–198,400	1168–151,844	1056–85,000	1009–149,747
Age group, n (%)						
Under 5	55 (85.94)	25 (37.35)	13 (20.63)	7 (14)	3 (6.82)	13 (21.70)
5–15	8 (12.5)	42 (62.65)	35 (55.56)	28 (56)	19 (43.18)	30 (50)
Adult	1 (1.56)	0 (0)	15 (23.81)	15 (30)	22 (50)	17 (28.30)
Haemoglobin g/dl (mean, SD)	ND	10.24 (1.90)	11.25 (1.90)	10.76 (1.92)	ND	10.43 (2.21)
Hb < 10 g/dl, n (%)	ND	29 (43.3)	14 (22.23)	16 (32)	ND	21 (35)

*ND* not done, *SD* standard deviation

The geometric mean of the parasite density at day 0 was 17,502 parasites/µl of blood (95% CI 13,000–22,003, range 1005–199,800). “The parasite density mean varied according to sites, from 7315 parasites/µl of blood in Ampasipotsy to 28,089 parasites/µl of blood in Tsaratanana”.

The combination was well tolerated. Few adverse events in gastrointestinal transit (vomiting, nausea, abdominal pain, anorexia), in the central nervous systems (headache, dizziness, drowsiness) and in musculoskeletal system were observed in 11.78% (41/348) patients, 2.58% (9/348) and 2.58% (9/348), respectively. The median haemoglobin concentration, among patients with available data (196/358), significantly increased from 10.60 g/dl on day 0 to 11.50 g/dl on day 28 (P < 0.00001).

The proportion of patients still harbouring parasites over the first 3 days were 42.65% (148/347) at day 1, 2.31% (8/346) at day 2 and 0% (0/340) at day 3, respectively.

The overall efficacy of ASAQ per protocol population analysis and after PCR correction was 99.70% (95% IC 98.30–99.95). According to years, PCR-corrected day 28 cure rates were 99.02% (95% IC 94.65–99.83) in 2012; 100% (95% IC 96.8–100) in 2013 and 100% (95% IC 96.65–100) in 2016. There was no case of ETF; 2 cases of LPF and one case of LCF were observed. The first patient classified as LPF was a 17-month old boy with a parasitaemia of 18,613 asexual parasites/µl at day 0 and 150,000 asexual parasites/µl at day 28. This patient was a true recrudescence as determined by *msp1/msp2* genotyping. The second patient classified as LPF was a 17-month old girl with a parasitaemia of 9900 asexual parasites/µl at day 0 and 29,232 asexual parasites/µl at day 28. The patient presenting a LCF, a 10-year old male, had a parasitaemia of 5811 asexual parasites/µl on day 0 and 9400 asexual parasites/µl on day 28 with 38.9 °C of axillary temperature. The latter two patients were classified as reinfection by *msp1/msp2* genotyping and were excluded from the analysis (Table 2).

**Table 2:** Treatment outcomes (per protocol analysis) by epidemiological strata, Madagascar 2012–2016, ASAQ

Epidemiological strata	Eastern Madagascar (equatorial stratum)			
Site	Matanga		Tsaratanana	
District	Vangaindrano		Ifanadiana	
Year	2012		2016	
Number of patients, n	64		67	
Clinical outcomes	Unadjusted-PCR	PCR-corrected	Unadjusted-PCR	PCR-corrected
ETF, n	0	0	0	0
LCT, n	0	0	0	0
LPF, n	2	1	0	0
ACPR, n (%)	62 (96.9)	62 (98.4)	65 (100)	65 (100)
CI 95% (%)	89.3–99.1	91.5–99.7	94.4–100	94.4–100
Withdrawal, n	0	1	2^a^	2^a^
Lost to follow-up, n	0	0	0	0

Epidemiological strata	Fringe highlands of Madagascar (fringe highlands stratum)			
Site	Ampasipotsy		Anjoma Ramartina	
District	Tsiroanomandidy		Mandoto	
Year	2012		2013	
Number of patients, n	44		60	
Clinical outcomes	Unadjusted-PCR	PCR corrected	Unadjusted-PCR	PCR corrected
ETF	0	0	0	0
LCT	1	0	0	0
LPF	0	0	0	0
ACPR	39 (97.5)	39 (100)	53 (100)	53 (100)
CI 95% (%)	87.1–99.6	91–100	93.2–100	93.2–100
Withdrawal, n	0	1	0	0
Lost to follow-up, n	4	4	7	7

Epidemiological strata	Western Madagascar (tropical stratum)			
Site	Ankazomborona		Antanimbary	
District	Marovoay		Maevatanana	
Year	2013		2016	
Number of patients, n	63		50	
Clinical outcomes	Unadjusted-PCR	PCR corrected	Unadjusted-PCR	PCR corrected
ETF	0	0	0	0
LCT	0	0	0	0
LPF	0	0	0	0
ACPR	63 (100)	63 (100)	46 (100)	46 (100)
CI 95% (%)	94.3–100	94.3–100	92.3–100	92.3–100
Withdrawal, n	0	0	0	0
Lost to follow-up, n	0	0	4	4

*ETF* early therapeutic failure, *LCF* late clinical failure, *LPF* late parasitological failure, *ACPR* adequate clinical and parasitological response

^a^Withdrawal of consent

## Discussion

Amodiaquine, in combination with artesunate, is widely used in sub-Saharan African countries, including Madagascar. The aim of this study was to provide updated data about the efficacy of ASAQ combination in Madagascar. Twelve years after its adoption as first-line treatment for acute uncomplicated falciparum malaria, ASAQ remains highly efficacious in the 6 geographical sites, covering various eco-epidemiological facies of the island, with different levels of malaria transmission. PCR-corrected cured rates estimated at day 28 were largely > 90%, above the WHO threshold for changing malaria treatment policy [5]. In addition, all patients treated with ASAQ cleared their parasites before day 3, indicating the absence of delayed parasite clearance, a marker for suspected partial resistance to artemisinin [8].

The proportion of cured patients observed in this study was not different from that reported in the two studies performed a decade earlier, suggesting the ASAQ retained its efficacy so far. These results are in line with those of comparable studies performed recently in neighbouring countries, such as Mozambique or Kenya [9, 10]. The results obtained in this study are also in line with those obtained in several African countries where ASAQ therapeutic failures are very rarely reported [11–18]. When documented, those therapeutic failures were not associated with the same *k13* mutations that were reported in *P. falciparum* isolates obtained during therapeutic failures in Cambodia and other Southeast Asian countries [19–22].

The treatment by ASAQ was well tolerated and no severe adverse event was reported among the participants in the 6 sites.

The efficacy of ASAQ, demonstrated in the present study, is necessary but not sufficient to contribute to the elimination of malaria in Madagascar. Stock out issues are recurrent on the island, emphasizing that sustainability of distribution of anti-malarials is crucial.

## Conclusion

ASAQ remains highly efficacious in the treatment of uncomplicated falciparum malaria in Madagascar. Thus, according to this study, the increased incidence of falciparum malaria cases observed recently seems not related to clinical treatment failure of first-line treatment promoted since 2006. Further investigations are required to determine the reasons for the recent increase in incidence of falciparum malaria.
